# Exploring Associations Between Frequency and Methods of Cannabis Use and Tooth Loss

**DOI:** 10.3290/j.ohpd.c_2829

**Published:** 2026-10-02

**Authors:** Ray M. Merrill

**Affiliations:** a Ray M. Merrill Professor, Brigham Young University, Department of Public Health, College of Life Sciences, Provo, UT, USA

**Keywords:** cannabis, marijuana, poor mental health, tobacco smoking, tooth loss.

## Abstract

**Purpose:**

The increasing use of cannabis for both medical and recreational purposes has been associated with negative effects on dental hygiene and oral health. This study examined the relationship between cannabis use and tooth loss.

**Materials and Methods:**

Analyses included 577,848 adults who participated in the 2016, 2018, 2020, 2022, and 2024 cross-sectional Behavioral Risk Factor Surveillance System (BRFSS) surveys. The association between past 30-day cannabis use and tooth loss was evaluated using multinomial logistic regression, adjusting for age, sex, race/ethnicity, survey year, tobacco smoking status, and 10 chronic conditions.

**Results:**

About 12.3% of participants reported using cannabis in the past 30 days, with 6.5% classified as infrequent users and 5.8% as daily or near-daily users. Smoking was the most common method, especially among daily or near-daily users. Cannabis use was more common among younger adults, men, non-Hispanic Black and multiracial adults, and current or former tobacco smokers. Use also increased over time, particularly among daily or near-daily users. Tooth loss increased with age and was more common among cannabis users, especially daily or near-daily users and those who smoked cannabis. Adults who smoked cannabis had a significantly higher prevalence of tooth loss than those using other consumption methods. After adjusting for factors such as age, sex, race/ethnicity, tobacco smoking, and 10 chronic conditions, daily or near-daily cannabis use remained significantly associated with greater odds of tooth loss, while infrequent use did not. Tooth loss was also strongly associated with chronic diseases, including arthritis, depression, asthma, and diabetes. Daily or near-daily users showed stronger associations with some chronic conditions, although no statistically significant association was found with heart attacks.

**Conclusion:**

Frequent and smoked cannabis use are associated with poorer oral health outcomes, particularly increased tooth loss.

Cannabis use has increased substantially in recent years,^18,24,27,28^ yet its potential impact on oral health outcomes remains incompletely understood. Although prior studies have linked cannabis consumption with periodontal disease, xerostomia, and other adverse oral conditions,^2,22,37^ evidence regarding its association with tooth loss is still limited, particularly when accounting for patterns of use, tobacco smoking status, and coexisting chronic disease. Given the growing prevalence of cannabis use in the United States and the increasing diversity of methods of consumption, understanding how frequency and method of use relate to oral health outcomes has important public health implications.

This study examined the relationship between past-30-day cannabis use and tooth loss in a nationally representative sample of adults. Cannabis use was categorized according to frequency of use, distinguishing infrequent users from daily or near-daily users, and further stratified by method of consumption, including smoked versus non-smoked cannabis. Demographic characteristics (age, sex, race/ethnicity), tobacco smoking status, and chronic disease burden were also evaluated to better characterize patterns of cannabis use and identify factors associated with tooth loss. In addition, the prevalence of common chronic diseases was assessed to determine whether these conditions were associated with tooth loss and may have confounded the observed associations.

The results provide evidence that daily or near-daily cannabis use, particularly smoked cannabis, is associated with increased odds of tooth loss after adjustment for several important demographic and behavioral risk factors. Patterns of cannabis use varied significantly by age, sex, race/ethnicity, and tobacco smoking status, with daily or near-daily users generally showing stronger associations with tooth loss. Furthermore, tooth loss was strongly associated with selected chronic conditions, and these relationships persisted even after adjustment for multiple covariates. Together, these findings highlight the importance of considering cannabis use behaviors, especially frequency and smoking-related exposure, in efforts to better understand and address oral health disparities among U.S. adults.

## MATERIALS AND METHODS

### Data

This study used cross-sectional data from the Behavioral Risk Factor Surveillance System (BRFSS). Administered to adults aged 18 and older across U.S. states, territories, and the District of Columbia (D.C.) (hereafter “states”), this nationwide telephone survey collects individual-level data on health behaviors, chronic conditions, and the use of preventive services. Primary strengths of the BRFSS encompass its robust sample size and the anonymity of its questionnaire, which together facilitate comprehensive and reliable public health analysis. The BRFSS questionnaire consists of three standardized parts: (1) a mandatory set of questions asked by all states, covering demographics and core health behaviors; (2) custom questions designed by individual states to address local health priorities; and (3) pre-approved Centers for Disease Control and Prevention (CDC) question sets that states can choose to include (e.g., cannabis use).^4^

The CDC added an optional cannabis module to the BRFSS in 2016, which numerous jurisdictions subsequently incorporated into their surveys. All states asked questions about dental visits and tooth loss, but only in even years. For the years 2016, 2018, 2020, 2022, and 2024, median response rates were 47%, 49%, 48%, 45%, and 44%, respectively.^5,6,7,8,9^ The study is based on 577,848 respondents who completed the cannabis module, after excluding 4885 individuals (0.84%) who refused to answer or did not know from the initial sample of 582,733 participants across 37 jurisdictions (33 states, D.C., and 3 territories).

Table 1 outlines the percentage of adults who reported cannabis use in the past 30 days, broken down by year and participant BRFSS jurisdiction. Participants in the areas where the cannabis module was offered represented 22% in 2016, 26% in 2018, 32% in 2020, 21% in 2022, and 30% in 2024 of all individuals completing the BRFSS survey. The number of years participating BRFSS areas adopted the optional cannabis module ranged from 1 to 4 years (mean = 2.4, SD = 0.92).

**Table 1:** Prevalence of past-month cannabis use by state using the Behavioral Risk Factor Surveillance System (BRFSS) cannabis module, by year

State		2016	2018	2020	2022	2024
	Unweighted n	% (SE)	% (SE)	% (SE)	% (SE)	% (SE)
Alaska	5,842	15.2 (1.1)	.	17.2 (1.0)	.	.
California	15,193	.	14.9 (0.5)	.	.	15.7 (0.7)
Colorado	12,700	13.6 (0.5)	.	.	.	.
Connecticut	13,476	.	.	.	16.0 (0.7)	15.0 (0.7)
Delaware	9,784	.	.	12.4 (0.9)	13.9 (1.0)	16.2 (1.1)
District of Columbia	2,633	.	.	.	.	19.9 (1.0)
Florida	44,263	7.4 (0.3)	10.0 (0.6)	.	.	.
Hawaii	19,630	.	.	11.4 (0.5)	11.8 (0.5)	13.6 (0.6)
Idaho	13,205	5.6 (0.5)	9.5 (0.8)	8.8 (0.6)	.	.
Illinois	16,276	.	.	10.9 (0.8)	15.6 (0.9)	16.6 (0.5)
Indiana	26,293	.	.	9.5 (0.5)	12.4 (0.5)	12.2 (0.4)
Kentucky	9,546	.	.	10.3 (0.7)	.	12.6 (0.7)
Maine	30,766	.	.	19.0 (0.7)	21.9 (0.7)	24.4 (0.6)
Maryland	28,312	.	7.9 (0.4)	9.2 (0.4)	.	.
Minnesota	56,672	6.5 (0.3)	8.9 (0.3)	10.3 (0.3)	.	16.8 (0.4)
Mississippi	17,119	5.9 (0.6)	.	9.2 (0.6)	11.4 (0.7)	10.4 (0.8)
Missouri	6,347	.	.	.	.	20.6 (0.7)
Montana	17,464	.	13.7 (0.7)	.	20.4 (0.7)	22.1 (0.7)
Nebraska	20,724	4.9 (0.4)	.	.	9.7 (0.6)	.
Nevada	4,572	.	.	.	19.5 (1.2)	20.2 (1.3)
New Hampshire	17,070	.	9.4 (0.7)	14.1 (0.8)	.	15.8 (0.8)
New Mexico	6,804	.	.	.	16.8 (0.9)	17.3 (1.1)
North Dakota	13,010	.	7.3 (0.6)	7.0 (0.6)	9.8 (0.7)	.
Ohio	34,758	7.2 (0.5)	9.4 (0.5)	12.1 (0.5)	.	.
Oregon	9,952	.	.	.	22.4 (0.8)	22.5 (0.7)
Rhode Island	9,135	.	.	15.8 (0.9)	.	19.7 (0.8)
South Carolina	20,727	.	8.6 (0.5)	9.4 (0.7)	.	10.7 (0.7)
Tennessee	13,114	5.5 (0.5)	9.5 (0.7)	9.3 (0.8)	.	.
Utah	9,715	.	.	8.4 (0.4)	.	.
Vermont	5,708	.	.	.	.	24.7 (0.8)
Virginia	12,889	.	.	.	13.1 (0.6)	13.8 (0.7)
West Virginia	15,308	.	6.9 (0.5)	8.3 (0.5)	.	12.2 (0.6)
Wisconsin	8,822	.	.	.	12.2 (0.5)	.
Wyoming	15,886	5.4 (0.7)	8.5 (0.6)	7.3 (0.7)	9.0 (0.7)	.
Guam	6,926	.	6.3 (1.1)	8.4 (1.0)	13.6 (1.6)	11.7 (3.6)
Puerto Rico	4,760	.	4.9 (0.5)	.	.	.
Virgin Island	2,447	.	.	.	10.4 (1.9)	10.7 (2.3)
Total	577,848	7.5 (0.2)	11.1 (0.2)	10.5 (0.2)	14.9 (0.2)	15.9 (0.2)
Data source: Centers for Disease Control and Prevention (CDC) Behavioral Risk Factor Surveillance System (BRFSS), 2018, 2020, 2022, 2024. Estimates were weighted to reflect the complex sample design. SE = Standard Error. Years without data are indicated by a dot (.)						

Oral consent was obtained from all participants during the initial screening. For an in-depth explanation of the BRFSS study design, survey instruments, and data collection procedures, please refer to the CDC BRFSS Frequently Asked Questions.^10^ This study was granted an exemption from human subjects review by the institutional review board, as it involved the secondary analysis of de-identified, publicly available BRFSS data.

### Measures

The primary outcome measured in this study was permanent tooth loss, based on the question “Not including teeth lost for injury or orthodontics, how many of your permanent teeth have been removed because of tooth decay or gum disease?” Responses were grouped into five categories: 0, 1–5, 6 or more but not all, all, and unknown.

Cannabis use within the preceding 30 days served as the primary independent variable. Cannabis use was assessed by the question: “During the past 30 days, on how many days did you use marijuana or cannabis?” While the BRFSS uses “marijuana” and “cannabis” interchangeably, this paper uses the scientifically precise term “cannabis” throughout. Participants were categorized into three groups based on their frequency of cannabis use in the past 30 days: non-users (0 days), infrequent users (1–19 days), and daily or near-daily users (20–30 days). Cannabis users were also asked “during the past 30 days, did you smoke it (for example, in a joint, bong, pipe, or blunt)?”

Participants were also categorized into four groups based on their cigarette smoking history: daily, occasional, former, and never smokers.

Covariates were selected a priori based on their established associations with cannabis use and tooth loss. Demographic covariates included age groups (18–24, 25–34, 35–44, 45–54, 55–64, and ≥ 65 years), sex (male, female), and race/ethnicity (non-Hispanic [NH] White, NH Black, NH Other, NH Multiracial, and Hispanic).

Additional covariates included survey year, tobacco-smoking status, and 10 common chronic conditions: asthma, arthritis, cancer, chronic obstructive pulmonary disease (COPD), coronary heart disease (CHD), depression, diabetes, heart attack, kidney disease, and stroke. The BRFSS assesses chronic conditions using self-reported data, specifically asking respondents if a doctor or other healthcare professional has ever diagnosed them with the condition.

### Model Specification

We used a multinomial logistic regression model to evaluate tooth loss status as the dependent variable. Tooth loss status has κ = 5 categories (none, 1-5, ≥ 6 but not all, all, unknown). Let P(y_i_ = k|x_i)_, where x_i_ represents the vector of cannabis (0, 1-19, 20-30) in the past 30 days and control variables for observation i. We are interested in modeling the log-odds of each tooth loss category relative to cannabis use. The log-odds of tooth loss category κ to not having lost any teeth are:


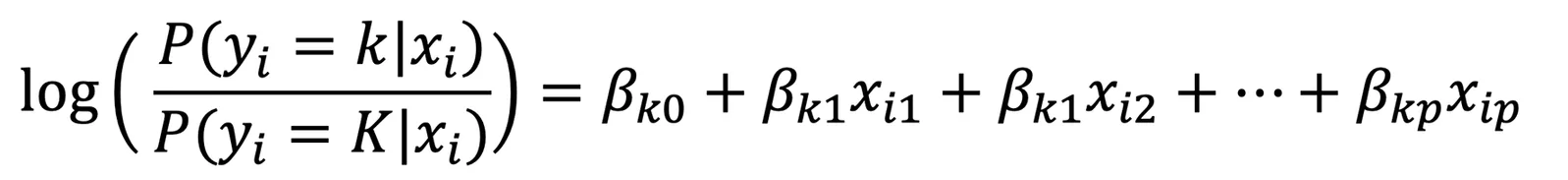


where:

P(y_i_ = k|x_i)_ is the probability that observation belongs to tooth loss category κ given its predictor values.

P(y_i_ = K|x_i)_ is the probability that observation belongs to the reference category given its predictor values.

β_k__0_ is the intercept for category κ (the baseline log-odds when all predictors are zero).

β_k__1_ is the coefficient for the predictor *j* in category κ (quantifies the effect of the predictor *j* on the log-odds of category κ relative to the reference).

x_i__1_ is the value of predictor for observation (the primary predictor is the cannabis use variable, and the other variables are the controls).

p is the total number of predictor variables (cannabis use, age, sex, race/ethnicity, year, smoking).

κ is the reference category, which is no tooth loss.

### Statistical Analysis

Categorical variables were summarized using frequencies and percentages, while continuous variables were reported as means and standard errors. All estimates were calculated using complex survey design elements, including strata, primary sampling units, and sampling weights. Standard errors were calculated using the Taylor series linearization method, adjusting for the complex sample design rather than assuming simple random sampling. We used multinomial logistic regression to evaluate the relationship between 30-day cannabis use and tooth loss status, adjusting for age, sex, race/ethnicity, survey year, and tobacco smoking status. Additional models further adjusted for the 10 chronic conditions. We estimated adjusted odds ratios and 95% confidence intervals; intervals excluding 1 indicated statistical significance. The significance of each independent variable within the regression models was assessed using a Type 3 analysis of effects, evaluating the unique contribution of each predictor while adjusting for the remaining variables. A p-value less than 0.05 was used to determine statistical significance. All statistical analyses were performed using SAS software, version 9.4 (SAS Institute; Cary, NC, USA).

## RESULTS

Table 2 presents the demographic and clinical characteristics of the study participants. Approximately 12.3% of people surveyed reported using cannabis during the past 30 days. Descriptive findings are presented as prevalence estimates, whereas associations with tooth-loss categories are presented as adjusted odds ratios from a multinomial logistic regression model. Most were infrequent users (1–19 days) at 6.5%, followed by daily or near-daily users (20–30 days) at 5.8%. Smoking was the most popular method, used by 76.7% of this group (69.5% among infrequent users and 84.7% among daily or near-daily users), while 23.3% consumed it in other ways. The diverse participant sample spanned all age groups and years, with a slight female majority. Most participants were never-smokers and non-Hispanic (NH) White.

**Table 2:** Adjusted odds ratios for tooth-loss categories according to past-30-day cannabis use

Variable	Unweighted n	Weighted % (SE)	1-5 (27.9%) vs 0 (55.8%)	6+, but not all (9.4%) vs 0 (55.8%)	All (5.0%) vs 0 (55.8%)	Unknown (1.8%) vs 0 (55.8%)	Type 3 F p-value
**Cannabis use**							
1-19 days	29,611	6.5 (0.08)	0.96 (0.90-1.02)	1.03 (0.94-1.12)	0.94 (0.83-1.07)	0.86 (0.64-1.17)	10.62
20-30 days	26,157	5.8 (0.07)	1.14 (1.07-1.22)	1.45 (1.32-1.58)	1.42 (1.26-1.61)	0.88 (0.69-1.13)	<0.0001
None	522,080	87.7 (0.10)	Reference	Reference	Reference	Reference	
**Age in years**							
18-24	31,088	11.1 (0.10)	Reference	Reference	Reference	Reference	
25-34	55,492	15.8 (0.11)	2.27 (2.09-2.45)	4.62 (3.50-6.09)	1.80 (1.14-2.82)	1.08 (0.74-1.58)	766.92
35-44	69,662	16.1 (0.11)	3.40 (3.14-3.67)	10.94 (8.36-14.31)	5.37 (3.46-8.32)	1.51 (1.05-2.16)	<0.0001
45-54	84,849	16.0 (0.11)	4.55 (4.21-4.92)	20.80 (15.94-27.15)	13.51 (8.80-20.77)	3.07 (2.15-4.39)	
55-64	116,188	17.2 (0.11)	6.54 (6.05-7.06)	48.49 (37.18-63.25)	39.08 (25.44-60.02)	6.12 (4.35-8.60)	
≥65	220,569	23.8 (0.11)	9.87 (9.14-10.65)	109.94 (84.38-143.24)	123.47 (80.33-189.77)	16.52 (11.95-22.84)	
**Sex**							
Men	261,010	48.4 (0.15)	Reference	Reference	Reference	Reference	
Women	316,720	51.6 (0.15)	1.02 (1.00-1.05)	1.13 (1.08-1.18)	1.25 (1.19-1.31)	1.17 (1.07-1.29)	14.00
Refused	118	0.0 (0.00)	1.20 (0.62-2.33)	1.10 (0.51-2.38)	5.11 (1.73-15.10)	3.72 (1.58-8.77)	<0.0001
**Race/Ethnicity**							
Non-Hispanic White	437,878	62.7 (0.15)	Reference	Reference	Reference	Reference	
Non-Hispanic Black	43,252	10.7 (0.09)	1.93 (1.84-2.02)	2.59 (2.44-2.76)	2.12 (1.94-2.31)	2.13 (1.87-2.43)	97.27
Non-Hispanic Other	31,081	7.2 (0.11)	1.33 (1.23-1.45)	1.24 (1.07-1.43)	0.85 (0.73-0.98)	2.08 (1.64-2.64)	<0.0001
Non-Hispanic Multiracial	12,798	1.9 (0.04)	1.21 (1.10-1.34)	1.54 (1.32-1.79)	1.16 (0.97-1.39)	1.13 (0.85-1.50)	
Hispanic	42,725	15.9 (0.13)	1.87 (1.78-1.96)	1.77 (1.62-1.92)	1.18 (1.03-1.35)	1.94 (1.58-2.38)	
Don’t know/refused	10,112	1.7 (0.03)	1.24 (1.12-1.38)	1.31 (1.13-1.52)	1.26 (1.06-1.49)	3.71 (3.03-4.53)	
**Survey year**							
2016	106,067	14.3 (0.07)	Reference	Reference	Reference	Reference	
2018	112,942	26.0 (0.10)	0.97 (0.93-1.01)	0.84 (0.79-0.89)	0.77 (0.71-0.83)	0.79 (0.69-0.90)	24.87
2020	129,223	19.1 (0.08)	0.92 (0.89-0.96)	0.75 (0.71-0.79)	0.87 (0.81-0.94)	1.03 (0.91-1.16)	<0.0001
2022	93,735	13.7 (0.07)	0.87 (0.84-0.91)	0.68 (0.64-0.72)	0.67 (0.62-0.73)	0.65 (0.57-0.73)	
2024	135,881	27.0 (0.10)	0.87 (0.84-0.91)	0.66 (0.62-0.71)	0.64 (0.60-0.69)	0.72 (0.63-0.83)	
**Tobacco smoking status**							
Daily	55,810	9.9 (0.08)	2.56 (2.43-2.69)	8.43 (7.90-8.99)	17.61 (16.29-19.03)	3.49 (3.02-4.04)	
Some days	20,880	4.1 (0.06)	1.81 (1.68-1.94)	3.91 (3.57-4.30)	7.25 (6.49-8.10)	2.43 (1.90-3.10)	496.97
Former	163,798	24.7 (0.12)	1.48 (1.44-1.54)	2.62 (2.50-2.75)	3.42 (3.23-3.63)	1.63 (1.47-1.81)	<0.0001
Never	333,808	60.7 (0.14)	Reference	Reference	Reference	Reference	
Don’t know/refused	3,552	0.6 (0.02)	1.33 (1.09-1.62)	1.57 (1.20-2.05)	2.98 (2.16-4.10)	5.50 (3.75-8.08)	
Data source: Centers for Disease Control and Prevention (CDC) Behavioral Risk Factor Surveillance System (BRFSS), 2016, 2018, 2020, 2022, 2024. Estimates were weighted to reflect the complex sample design. SE = Standard Error; OR= Odds Ratio; CI = Confidence Interval. Odds ratios were adjusted for age, sex, race/ethnicity, survey year, and tobacco smoking status.							

Cannabis use in the past 30 days declined statistically significantly with increasing age (Fig 1). The reduction was greater among daily or near-daily users than among infrequent users among adults aged 45 years and older. Men reported significantly higher cannabis use than women, particularly among daily or near-daily users compared with infrequent users. NH Black and NH multiracial adults had significantly higher odds of past-30-day cannabis use compared with NH White adults, with the largest differences observed among daily or near-daily users. Conversely, Hispanic and NH Other adults had significantly lower odds of past-30-day cannabis use compared with NH White adults. Past-30-day cannabis use increased significantly over time, with a greater increase observed among daily or near-daily users compared with infrequent users. The strongest associations were observed among current and former tobacco smokers, particularly for daily or near-daily cannabis use. Compared with never smokers, current daily smokers, current occasional smokers, and former smokers had statistically significantly higher odds of daily or near-daily cannabis use compared with infrequent use.

**Fig 1:**
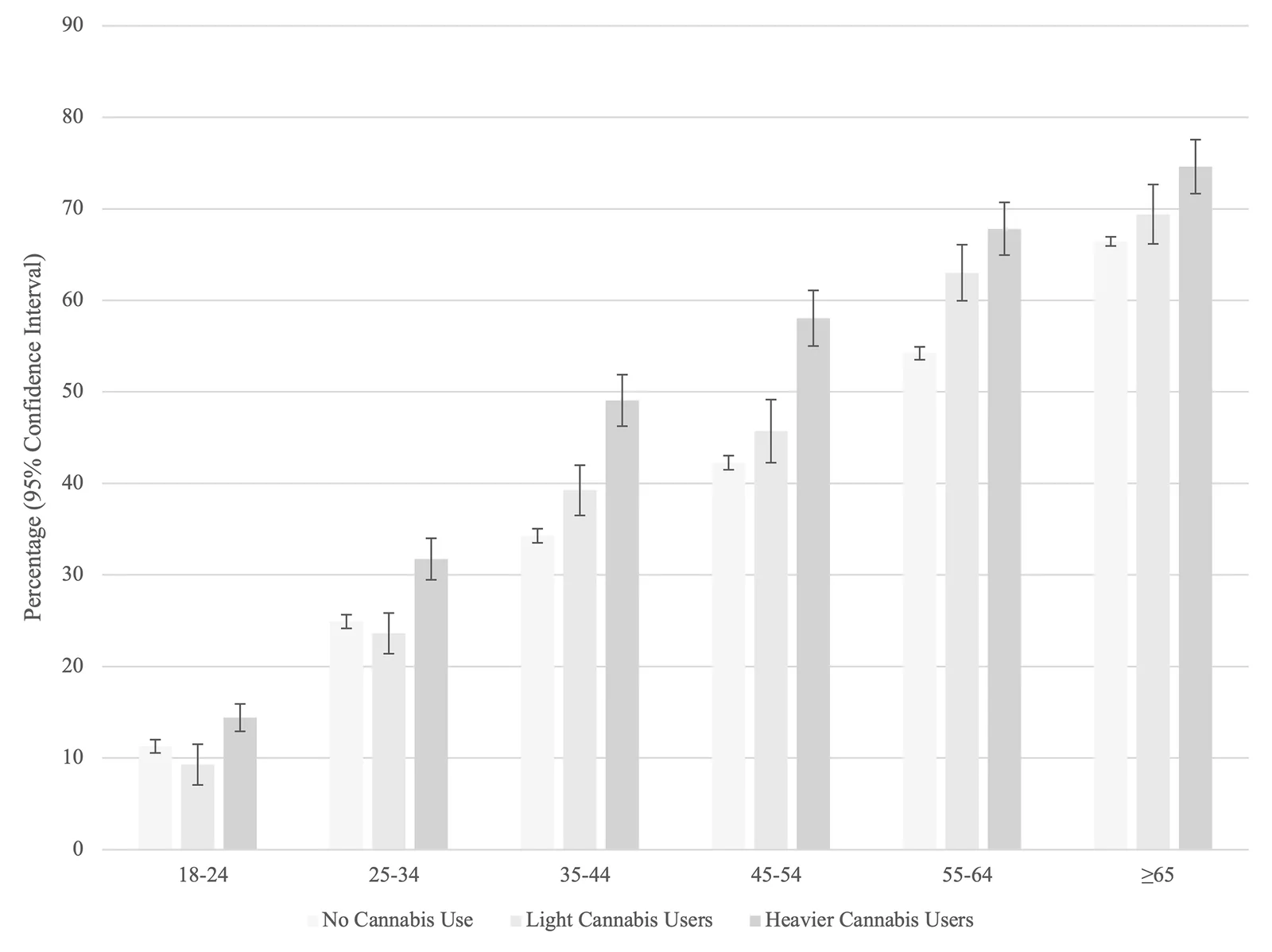
Percentage of participants who lost one or more teeth by age and cannabis use.

The distribution of tooth loss among participants was 55.8% (none), 27.9% (1-5), 9.4% (≥6 teeth, but not all), 5.0% (all), and 1.8% (unknown/refused). For respondents reporting tooth loss, the percentage with one or more missing teeth is presented by age and past 30day cannabis use (Fig 2). The percentage with one or more missing teeth increased with age and frequency of use. Among adults who used cannabis in the past 30 days, the prevalence of having one or more missing teeth increased with age and was significantly higher among those who smoked cannabis compared with those who used other methods of consumption across all age groups aged 25 years and older (Fig 3).

**Fig 2:**
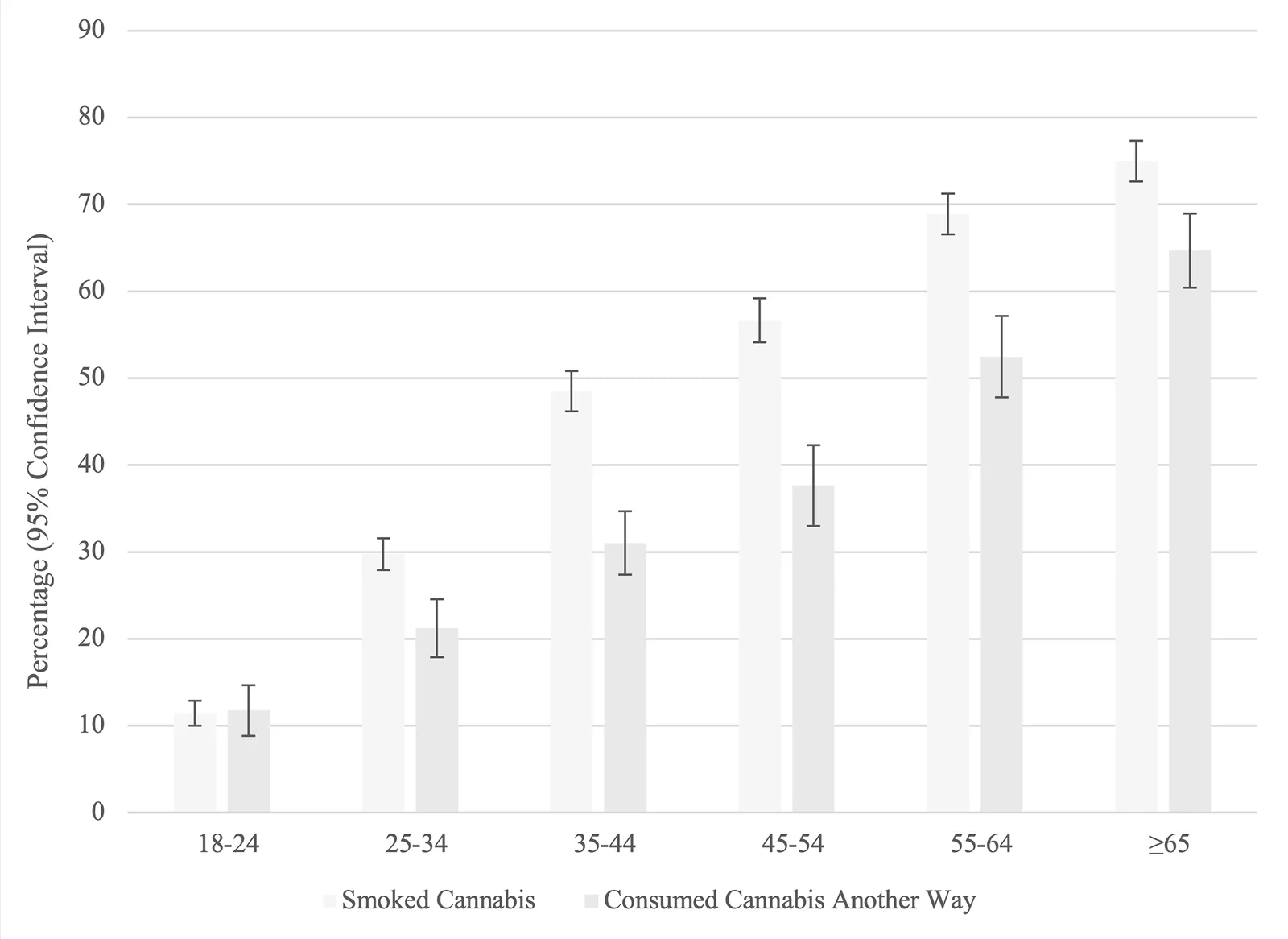
Percentage of past 30-day cannabis users who lost one or more teeth by age and method of consumption.

**Fig 3:**
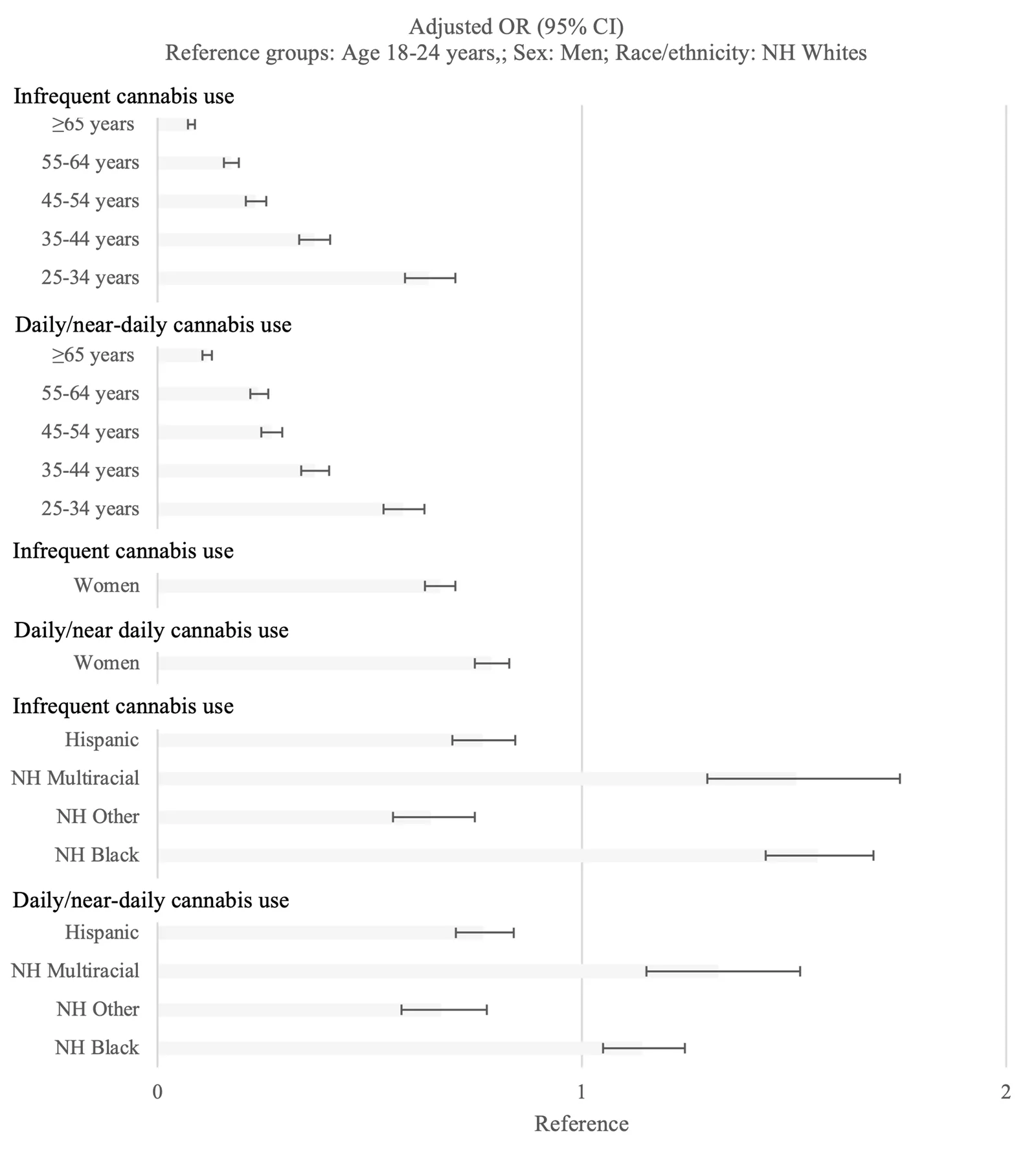

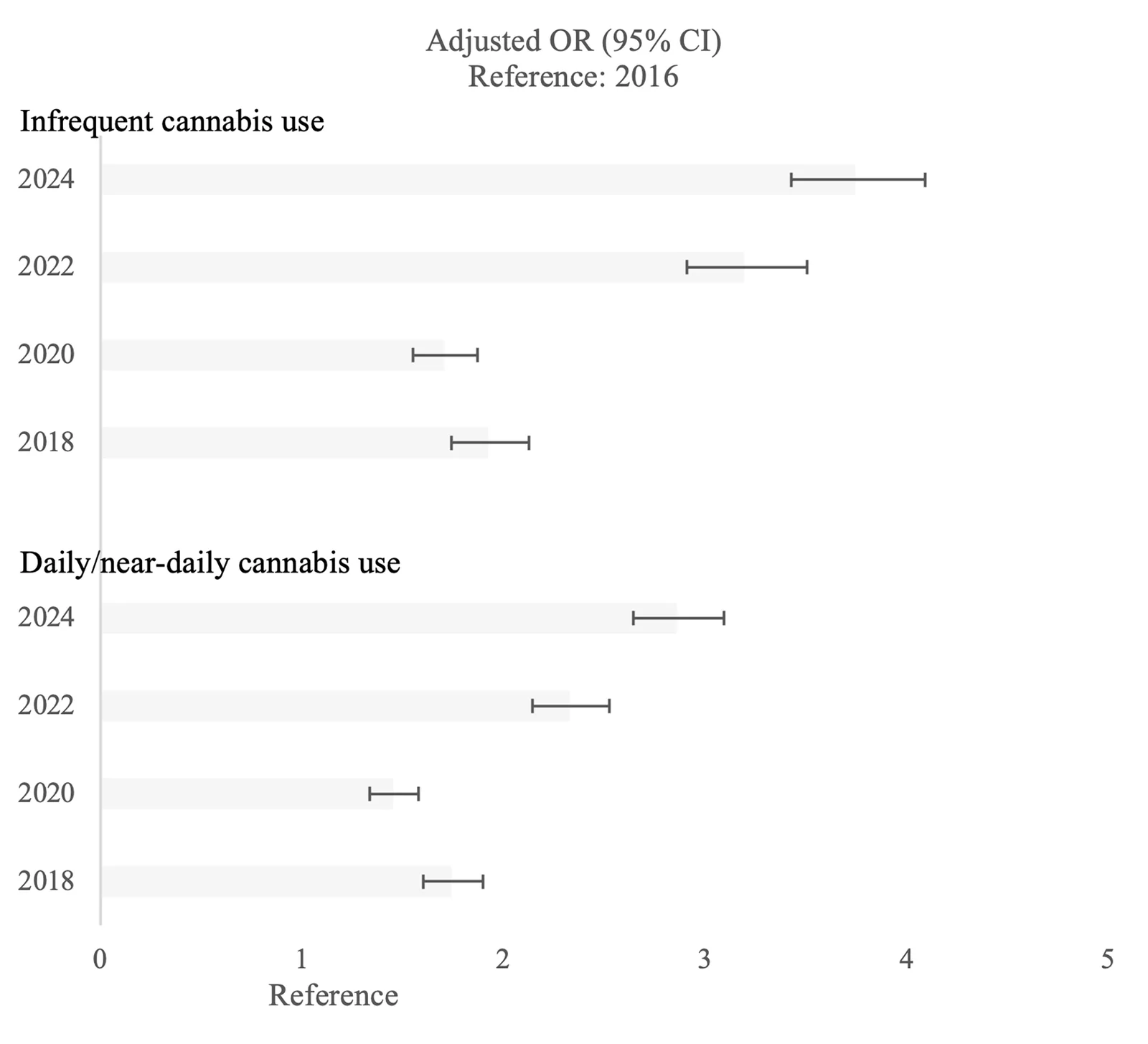

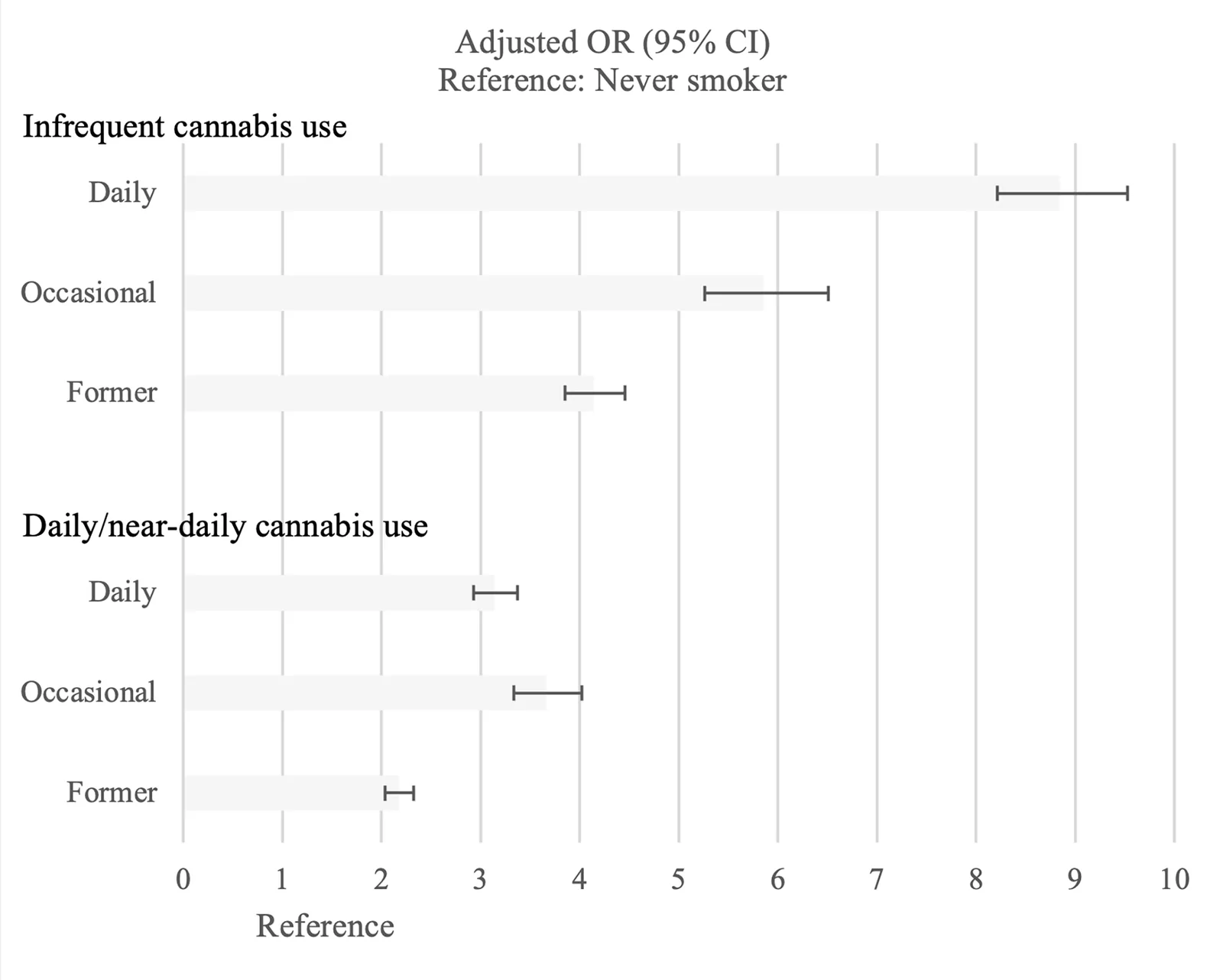
Adjusted odds ratios (95% confidence intervals) of past-30-day cannabis use by demographic variables. Odds ratios were estimated from a multivariable logistic regression model including age, sex, race/ethnicity, survey year, and cigarette smoking status simultaneously: Demographic characteristics (a), Survey year (b), Tobacco smoking status (c).

Table 2 presents both the prevalence distribution of tooth loss categories and the association between cannabis use and tooth loss estimated using regression models. Daily or near-daily cannabis use was associated with higher odds of tooth loss, even after accounting for factors like age, sex, race/ethnicity, and tobacco smoking. After covariate adjustment, infrequent cannabis use in the past month was not significantly associated with tooth loss compared to non-use.

Tooth loss was most strongly associated with age, smoking status, and race/ethnicity. Survey year, sex, cannabis use, and chronic disease were also significantly associated with tooth loss.

Table 3 presents the prevalence of 10 common chronic disease conditions among adults. Arthritis was the most frequently reported condition, followed by depression, asthma, and diabetes. Past-30-day cannabis use was positively associated with some chronic disease conditions, with the strongest associations observed among daily or near-daily users. In contrast, past-30-day cannabis use showed no statistically significant link to heart attacks and was less common among individuals with diabetes or prediabetes. Tooth loss was associated with all 10 chronic conditions, with increasing odds observed as the number of missing teeth increased. Tooth loss was positively associated with all chronic conditions, with increasing odds observed as the number of missing teeth increased.

**Table 3:** Adjusted odds ratios for past-30-day cannabis use and permanent tooth loss according to 10 common chronic conditions

Chronic disease	Unweightedn	Weighted% (SE)	Cannabis use in the past 30 days		Permanent tooth loss			
			1-19 days (6.5%) vs None (87.7%)	20-30 days (5.8%) vs None (87.7%)	1-5 (27.9%) vs 0 (55.3%)	6+, but not all (9.4%) vs 0 (55.3%)	All (5.0%) vs 0 (55,8%)	Unknown (1.8%) vs 0 (55.8%)
			OR (95% CI)	OR (95% CI)	OR (95% CI)	OR (95% CI)	OR (95% CI)	OR (95% CI)
**Heart attack**								
Yes	35,323	4.6 (0.06)	0.90 (0.79-1.02)	1.04 (0.93-1.17)	1.44 (1.34-1.54)	2.32 (2.16-2.50)	3.14 (2.90-3.41)	1.91 (1.62-2.26)
No	539,664	94.9 (0.06)	Reference	Reference	Reference	Reference	Reference	Reference
Don’t know/refused	2,861	0.5 (0.02)	0.73 (0.50-1.08)	0.67 (0.50-0.92)	2.05 (1.60-2.62)	3.35 (2.56-4.38)	3.46 (2.65-4.53)	7.13 (5.11-9.94)
CHD								
Yes	36,212	4.6 (0.05)	0.96 (0.84-1.10)	1.15 (1.00-1.31)	1.54 (1.44-1.64)	2.26 (2.11-2.44)	2.69 (2.47-2.93)	1.65 (1.44-1.88)
No	536,715	94.7 (0.06	Reference	Reference	Reference	Reference	Reference	Reference
Don’t know/refused	4,921	0.7 (0.02)	1.07 (0.76-1.50)	1.03 (0.79-1.34)	1.56 (1.30-1.88)	2.45 (2.01-3.00)	3.29 (2.66-4.08)	6.07 (4.50-8.17)
**Stroke**								
Yes	26,069	3.6 (0.05)	1.09 (0.94-1.26)	1.22 (1.06-1.41)	1.37 (1.26-1.48)	2.22 (2.03-2.43)	3.15 (2.88-3.46)	2.26 (1.86-2.74)
No	550,302	96.2 (0.05)	Reference	Reference	Reference	Reference	Reference	Reference
Don’t know/refused	1,477	0.2 (0.01)	1.02 (0.70-1.49)	1.51 (0.87-2.61)	1.38 (1.03-1.86)	2.43 (1.53-3.88)	2.89 (1.91-4.37)	6.12 (4.11-9.12)
**Asthma**								
Yes	81,788	14.6 (0.10)	1.24 (1.16-1.32)	1.48 (1.38-1.58)	1.11 (1.06-1.15)	1.53 (1.44-1.62)	1.79 (1.68-1.91)	1.15 (1.01-1.31)
No	494,279	85.1 (0.10)	Reference	Reference	Reference	Reference	Reference	Reference
Don’t know/refused	1,781	0.3 (0.02)	0.65 (0.42-1.01)	0.84 (0.56-1.26)	0.97 (0.75-1.26)	1.62 (1.16-2.27)	1.34 (0.95-1.89)	4.20 (2.99-5.90)
**COPD**								
Yes	49,166	7.0 (0.07)	1.09 (0.99-1.19)	1.43 (1.31-1.56)	1.48 (1.40-1.57)	2.84 (2.66-3.03)	4.14 (3.87-4.43)	2.26 (1.99-2.58)
No	526,238	92.6 (0.07)	Reference	Reference	Reference	Reference	Reference	Reference
Don’t know/refused	2,444	0.4 (0.02)	0.80 (0.58-1.11)	0.97 (0.70-1.35)	1.21 (0.94-1.55)	1.78 (1.37-2.32)	1.83 (1.37-2.44)	5.31 (3.62-7.81)
**Depression**								
Yes	113,033	19.5 (0.11)	1.86 (1.76-1.97)	2.20 (2.08-2.34)	1.28 (1.24-1.33)	1.99 (1.89-2.09)	2.19 (2.07-2.32)	1.48 (1.30-1.69)
No	462,224	80.0 (0.11)	Reference	Reference	Reference	Reference	Reference	Reference
Don’t know/refused	2,591	0.5 (0.02)	1.57 (1.11-2.22)	1.50 (1.08-2.07)	1.23 (0.98-1.55)	1.56 (1.20-2.04)	1.72 (1.18-2.51)	4.10 (3.13-5.38)
**Kidney**								
Yes	25,492	3.6 (0.05)	0.84 (0.73-0.97)	1.21 (1.04-1.40)	1.36 (1.26-1.48)	2.19 (1.98-2.42)	2.48 (2.25-2.73)	1.62 (1.34-1.97)
No	550,362	96.1 (0.06)	Reference	Reference	Reference	Reference	Reference	Reference
Don’t know/refused	1,994	0.3 (0.02)	0.97 (0.56-1.68)	0.82 (0.55-1.20)	1.44 (1.07-1.95)	2.70 (1.72-4.24)	3.23 (2.33-4.48)	5.65 (3.48-9.18)
**Cancer**								
Yes	62,711	7.8 (0.07)	1.10 (1.00-1.20)	1.32 (1.18-1.47)	1.11 (1.06-1.17	1.31 (1.22-1.39	1.35 (1.26-1.44	1.24 (1.08-1.43
No	513,226	91.9 (0.07)	Reference	Reference	Reference	Reference	Reference	Reference
Don’t know/refused	1,911	0.3 (0.02)	1.06 (0.66-1.70)	1.80 (1.28-2.52)	1.21 (0.91-1.62	2.27 (1.72-2.98	2.40 (1.76-3.28	4.65 (2.80-7.71
**Arthritis**								
Yes	204,099	27.6 (0.12)	1.20 (1.13-1.28)	1.53 (1.43-1.63	1.39 (1.35-1.44)	2.11 (2.02-2.21)	2.23 (2.12-2.35)	1.58 (1.45-1.74)
No	370,882	71.9 (0.12)	Reference	Reference	Reference	Reference	Reference	Reference
Don’t know/refused	2,867	0.5 (0.02)	1.20 (0.83-1.72)	1.11 (0.84-1.66)	1.55 (1.27-1.90)	2.19 (1.71-2.80)	2.11 (1.54-2.89)	6.69 (4.36-10.24)
**Diabetes**								
Yes	82,396	12.3 (0.10)	0.75 (0.67-0.83)	0.75 (0.68-0.83)	1.49 (1.42-1.56)	2.26 (2.14-2.39)	2.70 (2.54-2.87)	1.76 (1.57-1.98)
Yes, during pregnancy	4,969	1.0 (0.03)	0.82 (0.65-1.03)	0.92 (0.68-1.25)	1.05 (0.91-1.21)	1.09 (0.88-1.35)	0.91 (0.67-1.24)	1.50 (0.96-2.36)
No	476,032	84.1 (0.11)	Reference	Reference	Reference	Reference	Reference	Reference
**Prediabetes**	13,500	2.4 (0.05)	0.78 (0.66-0.93)	0.88 (0.71-1.10)	1.39 (1.26-1.53)	2.07 (1.80-2.39)	1.54 (1.34-1.77)	1.44 (1.05-1.96)
Don’t know/refused	951	0.2 (0.01)	0.81 (0.44-1.48)	0.61 (0.35-1.07)	1.09 (0.79-1.51)	2.16 (1.41-3.32)	2.26 (1.36-3.76)	6.50 (4.28-9.87)
Data source: Centers for Disease Control and Prevention (CDC) Behavioral Risk Factor Surveillance System (BRFSS), 2016, 2018, 2020, 2022, 2024. Estimates were weighted to reflect the complex sample design. CHD = Coronary Heart Disease; COPD = Chronic Obstructive Pulmonary Diseases; OR = Odds Ratio; CI = Confidence Interval. All Odds Ratios (ORs) were adjusted for age, sex, race/ethnicity, survey year, and tobacco smoking status.								

Table 4 presents adjusted odds ratios for the association between cannabis use and tooth loss after accounting for chronic conditions. After adjustment for the 10 chronic conditions, daily or near-daily cannabis use remained significantly associated with all categories of tooth loss compared with non-use. Conversely, infrequent cannabis use was not significantly associated with tooth loss (Table 4).

**Table 4:** Adjusted odds ratios for tooth loss according to past-30-day cannabis use, additionally adjusted for 10 chronic conditions

	Weighted n	Weighted % (SE)	1-5 (28.0%) vs 0 (55.8%)	6+, but not all (9.4%) vs 0 (55.8%)	All (5.0%) vs 0 (55.8%)	Unknown (1.8%) vs 0 (55.8%)	
			OR (95% CI)	OR (95% CI)	OR (95% CI)	OR (95% CI)	Type 3 F p-value
Cannabis use							
1-19 days	29,618	6.5 (0.08)	0.94 (0.88-1.01)	0.99 (0.90-1.08)	0.91 (0.80-1.03)	0.85 (0.62-1.15)	6.51
20-30 days	26,157	5.8 (0.07)	1.11 (1.04-1.18)	1.32 (1.20-1.45)	1.30 (1.14-1.47)	0.85 (0.66-1.09)	<0.0001
None	522,073	87.7 (0.10)	Reference	Reference	Reference	Reference	
Data source: Centers for Disease Control and Prevention (CDC) Behavioral Risk Factor Surveillance System (BRFSS), 2016, 2018, 2020, 2022, 2024. Estimates were weighted to reflect the complex sample design. OR = Odds Ratio; CI = Confidence Interval. Odds Ratios were adjusted for age, sex, race/ethnicity, survey year, tobacco smoking status, and 10 chronic diseases (heart attack, CHD [CHD], stroke, asthma, chronic obstructive pulmonary disease [COPD], depression, kidney disease, arthritis, diabetes, and cancer).							

Past-30-day cannabis users were further categorized according to whether they smoked cannabis (Table 5). Compared with infrequent users, daily or near-daily cannabis use was significantly associated with higher odds of tooth loss; compared with users who reported only non-smoking methods, smoked cannabis use was also significantly associated with higher odds of tooth loss. Furthermore, the magnitude of these associations increased with greater severity of tooth loss.

**Table 5:** Adjusted odds ratios for tooth-loss categories versus no tooth loss among adults who reported cannabis use in the past 30 days, according to cannabis-use frequency and smoking status

	Weighted n	Weighted % (SE)	1-5 (28.0%) vs 0 (55.8%)	6+, but not all (9.4%) vs 0 (55.8%)	All (5.0%) vs 0 (55.8%)	Unknown (1.8%) vs 0 (55.8%)	
			OR (95% CI)	OR (95% CI)	OR (95% CI)	OR (95% CI)	Type 3 F p-value
Cannabis use/smokes it							
20-30 days/yes	21,830	40.1 (0.43)	1.41 (1.23-1.62)	2.27 (1.82-2.82)	2.35 (1.67-3.29)	1.91 (1.20-3.04)	7.21
1-19 days/yes	20,316	36.6 (0.43)	1.26 (1.10-1.44)	1.84 (1.48-2.29)	1.85 (1.31-2.62)	2.34 (1.40-3.90)	<0.0001
20-30 days/no	4192	7.2 (0.23)	1.14 (0.94-1.38)	1.46 (1.09-1.96)	1.63 (1.06-2.50)	1.25 (0.69-2.27)	
1-19 days/no	9070	16.1 (0.32)	Reference	Reference	Reference	Reference	
Data Source: Centers for Disease Control and Prevention (CDC) Behavioral Risk Factor Surveillance System (BRFSS), 2016, 2018, 2020, 2022, 2024. Estimates were weighted to reflect the complex sample design. OR = Odds Ratio. CI = Confidence Interval. Odds Ratios were adjusted for age, sex, race/ethnicity, survey year, tobacco smoking status, and 10 chronic diseases (heart attack, coronary heart disease [CHD], stroke, asthma, chronic obstructive pulmonary diseases [COPD], depression, kidney disease, arthritis, diabetes, and cancer). This table excludes 367 cannabis users with missing consumption method data.							

## DISCUSSION

This study examined the demographic distribution of past-30-day cannabis use, patterns of consumption, and associations with tooth loss while accounting for 10 chronic conditions in a diverse adult population. Several important findings emerge. First, cannabis use was relatively uncommon overall (12.3%), but among users there was a nearly even split between infrequent and daily or near-daily use, indicating that daily or near-daily use represented a substantial proportion of cannabis use. Second, smoking was the dominant mode of use, particularly among daily or near-daily users. Third, cannabis use showed clear demographic gradients by age, sex, and race/ethnicity, and increased over time. Finally, and most importantly, daily or near-daily cannabis use—especially when smoked—was associated with greater odds of tooth loss even after adjustment for sociodemographic factors, tobacco use, and multiple chronic conditions. Adjustment for these factors was important because prior epidemiologic studies have identified age, sex, race/ethnicity, tobacco use, and chronic conditions as factors associated with both cannabis use and tooth loss, making them potential sources of confounding when evaluating associations between cannabis use and oral health.^13,16,19,26,29,32,38^

A key pattern in these findings is the strong age-related gradient in cannabis use, with the steepest reduction observed among daily or near-daily users. This likely reflects cohort differences in attitudes toward cannabis, generational variation in exposure and legalization contexts, and potentially differential survival or health selection effects among older adults.^3,15,17,35^ The consistently lower prevalence of cannabis use in older age groups is notable given increasing legalization and normalization of cannabis use in recent years, suggesting that behavioral patterns established earlier in life may continue to shape patterns of cannabis use across age groups and survey years. The strong association between age and tooth loss observed in this study is consistent with previous research demonstrating that tooth loss increases substantially with age.^26,29^

Sex and racial/ethnic disparities were also evident. Men had a higher prevalence of past-30-day cannabis use than women, particularly at daily or near-daily levels of consumption, consistent with broader substance use epidemiology.^19^ More striking were racial/ethnic differences: NH Black and NH multiracial adults exhibited higher odds of past-30-day cannabis use, especially daily or near-daily use, while Hispanic and other NH groups showed lower odds compared to NH White adults. These disparities in cannabis use likely reflect a combination of structural factors, including differential exposure to stressors such as discrimination and minority stress, variation in neighborhood-level availability and enforcement practices, and cultural differences in substance use norms, all of which have been shown to shape patterns of cannabis use across populations.^14,20,30,31,33^ The stronger disparities observed for daily or near-daily use suggest that inequities may become more pronounced as consumption intensity increases. These demographic differences are also relevant to oral health, as previous studies have documented racial/ethnic and sex-related disparities in tooth loss.^26,29^ Thus, the observed association between cannabis use and tooth loss should be interpreted in the context of these overlapping demographic patterns.

The observed increase in cannabis use over time aligns with broader societal shifts, including legalization trends and changing perceptions of risk.^18,24,27,28^ Importantly, the increase was more pronounced among daily or near-daily users, indicating that the time trend differed by frequency category. This pattern raises concerns about potential health consequences, particularly given the associations observed with oral health outcomes.

A particularly robust finding in this study is the strong association between tobacco use and cannabis use, as observed previously.^32^ Current and former tobacco smokers had a significantly higher prevalence of past-30-day cannabis use, especially daily or near-daily use. This clustering of behaviors suggests shared risk pathways, co-dependence, or overlapping social and environmental influences.^1^ The gradient observed across never, former, and current smokers further supports the possibility of behavioral reinforcement between tobacco and cannabis use (e.g., situations like socializing, stress, or relaxation can trigger both substances and exposure to one can increase the desire for the other). This co-use is especially important in the context of oral health, as tobacco is a well-established risk factor for periodontal disease and tooth loss, with evidence demonstrating a dose-response relationship between smoking exposure and tooth loss.^2,16,22,37^ However, the association between cannabis use and tooth loss did not differ significantly by smoking status (F p = 0.0764). The persistence of the association between daily or near-daily cannabis use and tooth loss after adjustment for smoking suggests that the observed relationship was not fully attenuated by tobacco use.

The central finding of this study is the association between daily or near-daily cannabis use and increased odds of tooth loss, even after adjustment for sociodemographic characteristics, tobacco smoking, and multiple chronic diseases. Importantly, infrequent cannabis use was not statistically significantly associated with tooth loss after adjustment, suggesting a frequency-related gradient rather than a simple binary effect. This gradient strengthens the plausibility of a causal association and suggests that oral health risks may be concentrated among frequent users rather than occasional consumers.

The method of cannabis consumption may also play an important role in this association. Smoking cannabis was strongly associated with higher odds of tooth loss, and this relationship became more pronounced with increasing severity of tooth loss. This finding suggests that combustion-related exposure may contribute to tooth loss, potentially through mechanisms such as periodontal inflammation, xerostomia (dry mouth), changes in oral microbiota, and reduced salivary flow.^11,23,25^ Additionally, smoked cannabis is often used in conjunction with tobacco or in similar behavioral contexts, which may further exacerbate oral health risks. This finding is also consistent with previous studies suggesting that cannabis smoking may increase the risk of periodontal disease independently of tobacco smoking.^34^

Tooth loss itself demonstrated a strong and consistent relationship with age and the 10 chronic conditions, as expected.^18,19,22^ However, cannabis use remained significantly associated with tooth loss even after accounting for these conditions. This indicates that cannabis use remained associated with tooth loss after adjustment for the measured covariates, or may be part of a broader clustering of health-risk behaviors. The observed associations between tooth loss and chronic conditions should be interpreted cautiously, as the cross-sectional design does not allow determination of whether chronic diseases preceded tooth loss, resulted from oral health deterioration, or share common underlying risk factors. The strong associations between tooth loss and multiple chronic conditions—including arthritis, diabetes, asthma, and depression—may reflect shared pathways involving inflammation, medication use, health behaviors, socioeconomic factors, and access to healthcare rather than direct causal relationships.. These findings are consistent with prior research highlighting bidirectional relationships between oral disease and chronic conditions through inflammatory and behavioral pathways.^12,21,36^ Previous research has identified associations between chronic disease and tooth loss; however, the mechanisms linking specific medical conditions with oral health outcomes remain complex and are likely multifactorial.^38^

The relationship between cannabis use and chronic disease was also notable. This finding is consistent with previous reports showing that adults with chronic medical conditions are more likely to use cannabis, often for symptom management.^13^ However, the observed associations between cannabis use and chronic conditions in this study should not be interpreted as evidence of causality. Recent cannabis use was positively associated with a range of chronic conditions, particularly among daily or near-daily users. These associations may reflect reverse causation, whereby individuals with chronic conditions use cannabis to manage symptoms, as well as shared behavioral, socioeconomic, and health-related factors that contribute to both cannabis use and chronic disease. The lack of association with heart attack and the lower prevalence of cannabis use among individuals with diabetes or prediabetes further suggest that relationships may vary by disease type and severity.

Several limitations should be considered when interpreting these findings. The cross-sectional design precludes causal inference and raises the possibility of reverse causation, particularly in the relationship between cannabis use and chronic disease. Self-reported measures of cannabis use and tooth loss may introduce recall or reporting bias. Additionally, residual confounding is possible, particularly from unmeasured variables such as oral hygiene practices, diet, access to dental care, and socioeconomic status. Changes in cannabis legalization and social acceptability over time may also influence reporting behavior and observed trends. Finally, although current cannabis use may reflect a longer history of consumption, information on past use—which may be a stronger predictor of tooth loss—was unavailable. Therefore, these findings should be considered exploratory, and longitudinal studies are needed to more directly assess the relationship between cannabis use and tooth loss.

Despite these limitations, the study has several strengths, including a large and demographically diverse sample, detailed categorization of cannabis use frequency and method of consumption, and adjustment for multiple potential confounders including tobacco use and 10 chronic conditions. The consistent frequency-related patterns observed across analyses and the consistency of the association between daily or near-daily smoked cannabis use and tooth loss support the findings.

## CONCLUSION

This study suggests that daily or near-daily cannabis use—particularly when smoked—is associated with increased odds of tooth loss after adjustment for key demographic, behavioral, and clinical factors. The findings highlight the importance of considering oral health outcomes in the context of increasing cannabis use and underscore the need for public health messaging that addresses not only frequency of use but also mode of consumption. Future longitudinal research is needed to clarify causal pathways and to better understand the mechanisms linking cannabis use to oral and systemic health outcomes.

## References

[ref23] Manas D, Patel N (2025). The clinical implications of cannabis use in dentistry. BDJ Team.

[ref31] Rafei P, Englund A, Lorenzetti V, Elkholy H, Potenza MN, Baldacchino AM (2023). Transcultural aspects of cannabis use: A descriptive overview of cannabis use across cultures. Curr Addict Rep.

[ref34] Thomson WM, Poulton R, Broadbent JM, Moffitt TE, Caspi A, Beck JD (2008). Cannabis smoking and periodontal disease among young adults. JAMA.

